# Recovery of Red Fluorescent Protein Chromophore Maturation Deficiency through Rational Design

**DOI:** 10.1371/journal.pone.0052463

**Published:** 2012-12-20

**Authors:** Matthew M. Moore, Samuel K. Oteng-Pabi, Antonia T. Pandelieva, Stephen L. Mayo, Roberto A. Chica

**Affiliations:** 1 Division of Biology, California Institute of Technology, Pasadena, California, United States of America; 2 Department of Chemistry, University of Ottawa, Ottawa, Ontario, Canada; 3 Division of Chemistry and Chemical Engineering, California Institute of Technology, Pasadena, California, United States of America; 4 Centre for Catalysis Research and Innovation, University of Ottawa, Ottawa, Ontario, Canada; Weizmann Institute of Science, Israel

## Abstract

Red fluorescent proteins (RFPs) derived from organisms in the class Anthozoa have found widespread application as imaging tools in biological research. For most imaging experiments, RFPs that mature quickly to the red chromophore and produce little or no green chromophore are most useful. In this study, we used rational design to convert a yellow fluorescent mPlum mutant to a red-emitting RFP without reverting any of the mutations causing the maturation deficiency and without altering the red chromophore’s covalent structure. We also created an optimized mPlum mutant (mPlum-E16P) that matures almost exclusively to the red chromophore. Analysis of the structure/function relationships in these proteins revealed two structural characteristics that are important for efficient red chromophore maturation in DsRed-derived RFPs. The first is the presence of a lysine residue at position 70 that is able to interact directly with the chromophore. The second is an absence of non-bonding interactions limiting the conformational flexibility at the peptide backbone that is oxidized during red chromophore formation. Satisfying or improving these structural features in other maturation-deficient RFPs may result in RFPs with faster and more complete maturation to the red chromophore.

## Introduction

Red fluorescent proteins (RFPs) derived from organisms in the class Anthozoa are widely employed as imaging tools in biological research. For example, these proteins have been used as markers of gene expression, expressed as fusion partners for the tracking of intracellular endogenous protein-RFP chimeras, and complemented with other fluorescent proteins (FPs) for use in fluorescence resonance energy transfer experiments [1], [2]. The longer emission wavelengths of RFPs result from a chromophore whose *p*-hydroxybenzylidene-imidazolinone group is prepended with an acylimine substituent at the C1 atom as a result of backbone oxidation [3], [4]. The extended conjugation afforded by unsaturation from the acylimine lowers the energy of both excitation and emission from the analogous fluorophore found in green fluorescent proteins (GFPs), resulting in a bathochromic wavelength shift [5].

Chromophore maturation in RFPs is a long-studied phenomenon. In the past, green chromophore maturation was seen as an intermediate step on the pathway to complete red chromophore maturation [6]; this interpretation turned out to be incorrect [7]. Recent work has revealed much about how the red and green chromophore pathways branch off from one another during the maturation process. A study using deuterium labeling uncovered evidence that a branch upstream to green chromophore formation splits the population of proteins in solution into two: a population that will mature to the GFP-like green chromophore and one that will pass through a blue intermediate species to form the RFP red chromophore [7]. Previous to this study, characterization of the RFP HcRed revealed a peak at 410 nm during maturation that corresponds to this blue intermediate species [8]. This peak is significant in that it shows the expected behavior of an intermediate species in the red chromophore maturation process. In addition, X-ray crystallography and computational modeling support the existence of a species with the structure that has been proposed for this blue intermediate [9], [10].

**Figure 1:**
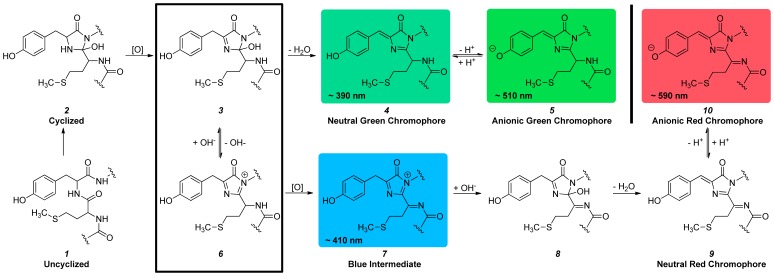
Chromophore maturation mechanism. The chromophore maturation mechanism proposed by Strack et al. [7] is a branched pathway ending with either red or green chromophore formation. Intermediates and final products on this pathway that are observable by absorption spectroscopy are color-coded and labeled with the approximate wavelength of their peak absorption. The branch point is indicated by a box.

A schematic illustrating the proposed mechanism for chromophore formation in RFPs is shown in Figure 1. Briefly, the uncyclized chromogenic tripeptide (Xaa-Tyr-Gly) ***1*** undergoes a cyclization reaction that is trapped by oxidation to a colorless intermediate species ***3***. This colorless intermediate reversibly eliminates a hydroxide moiety yielding ***6***, thus enabling a branch in the chromophore maturation pathway. Colorless intermediates that eliminate hydroxide are poised to undergo further oxidation, resulting in the crystallographically observable blue intermediate with an absorption peak at 410 nm (***7***) that was mentioned above. The exact electronic structure of this blue intermediate is unknown–both cationic [7] and anionic [10] structures have been proposed. Completion of this second oxidation reaction seals the fate of FPs and directs their maturation to the red chromophore (***9*** and ***10***). A base-induced elimination of water follows this second oxidization as evidenced by a kinetic isotope effect on the C-beta carbon of the conserved tyrosine residue central to the chromogenic tripeptide [7]. Colorless intermediates that retain hydroxide (***3***) may also undergo elimination of water by proton abstraction at the C-beta carbon of the conserved tyrosine residue, leading to production of FPs with green chromophores (***4*** and ***5***) in the other branch of this maturation pathway [11].

Red and green chromophores cannot interconvert–both are dead-end products of chromophore maturation [6], [7]. Consequently, some RFPs express as mixtures of proteins with either green or red chromophores. DsRed and its mutant mPlum are notable examples of RFPs that express in this fashion [12], [13]. Studies of Lys70 in various mutants of DsRed indicate that this residue is crucial for the formation of the acylimine group and thus red fluorescence [3], [13]. Additionally, the proximity of the Lys70 terminal amino group to the chromophore ring system has been shown to correlate with increasing quantum yield (Φ_F_) among DsRed mutants [4], [14].

Fast and complete maturation to the red chromophore is a desired property for RFPs used in imaging experiments. To improve maturation efficiency, investigators have used directed evolution and have obtained RFPs that display fast and efficient red chromophore maturation such as mCherry [15], DsRed.T4 [16], and mKate2 [17]. Here, we present a rational approach to enhance RFP chromophore maturation in the DsRed mutant mPlum. We hypothesized that the identity of the amino acid at position 16 may be crucial to promoting or inhibiting red chromophore maturation. Because residues at position 16 interact directly with the peptide bond that oxidizes during red chromophore formation, we postulated that this interaction could interfere with the oxidation reaction at this position. Using rational design, we converted a yellow-emitting mPlum mutant to a red-emitting RFP by replacing Glu16 with small non-polar amino acids. We also created an optimized mPlum mutant (mPlum-E16P) that matures almost exclusively to the red chromophore.

## Results and Discussion

### Red Chromophore Maturation Deficient mPlum Mutant

In a previous study, we developed a structure-based, rational design approach that combined computational protein design and experimental screening of combinatorial mutant libraries to red shift the emission wavelength of RFPs [18]. One of the hypotheses that we formulated to achieve this goal was that the introduction of a π-stacking interaction with the chromophore phenol ring (similar to the one observed in yellow fluorescent proteins [19]) would red shift the emission wavelength of mCherry by stabilizing the excited state. Using our combined computational and experimental approach, we successfully introduced a π-stacking interaction with the mCherry chromophore by making a I197Y mutation (numbering based on DsRed). Two additional mutations, T195A and A217C, improved the quantum yield of the single mutant I197Y. This mutational motif, termed “AYC” for A195/Y197/C217, resulted in a 10 nm bathochromic shift in emission wavelength relative to mCherry [18].

To create a further red-shifted monomeric RFP, we investigated whether the AYC motif could red shift the emission wavelength of the far-red emitting RFP mPlum [20]. mPlum is a member of the mFruit family of monomeric RFPs derived from DsRed [15] and differs from mCherry by 13 mutations (excluding N- and C-terminal tags). Its fluorescence emission wavelength (λ_em_) of 649 nm is the longest of the mFruits and is red-shifted 38 nm compared to that of mCherry (λ_em_ = 611 nm). Ile65 and Glu16 appear to be major contributors to mPlum’s long emission wavelength as replacement of either residue results in an up to 40 nm hypsochromic shift [20]. These residues interact through an H-bond between the side chain of Glu16 and the acylimine carbonyl oxygen atom of Ile65 [21], and this interaction gives rise to a dynamic Stokes shift that is responsible for the far-red emission wavelength of 649 nm [12]. Given the very high sequence identity between mCherry and mPlum (>90%), we hypothesized that the AYC motif would red shift the emission wavelength of mPlum as was observed in mCherry, yielding a monomeric RFP with λ_em_ >650 nm.

**Figure 2:**
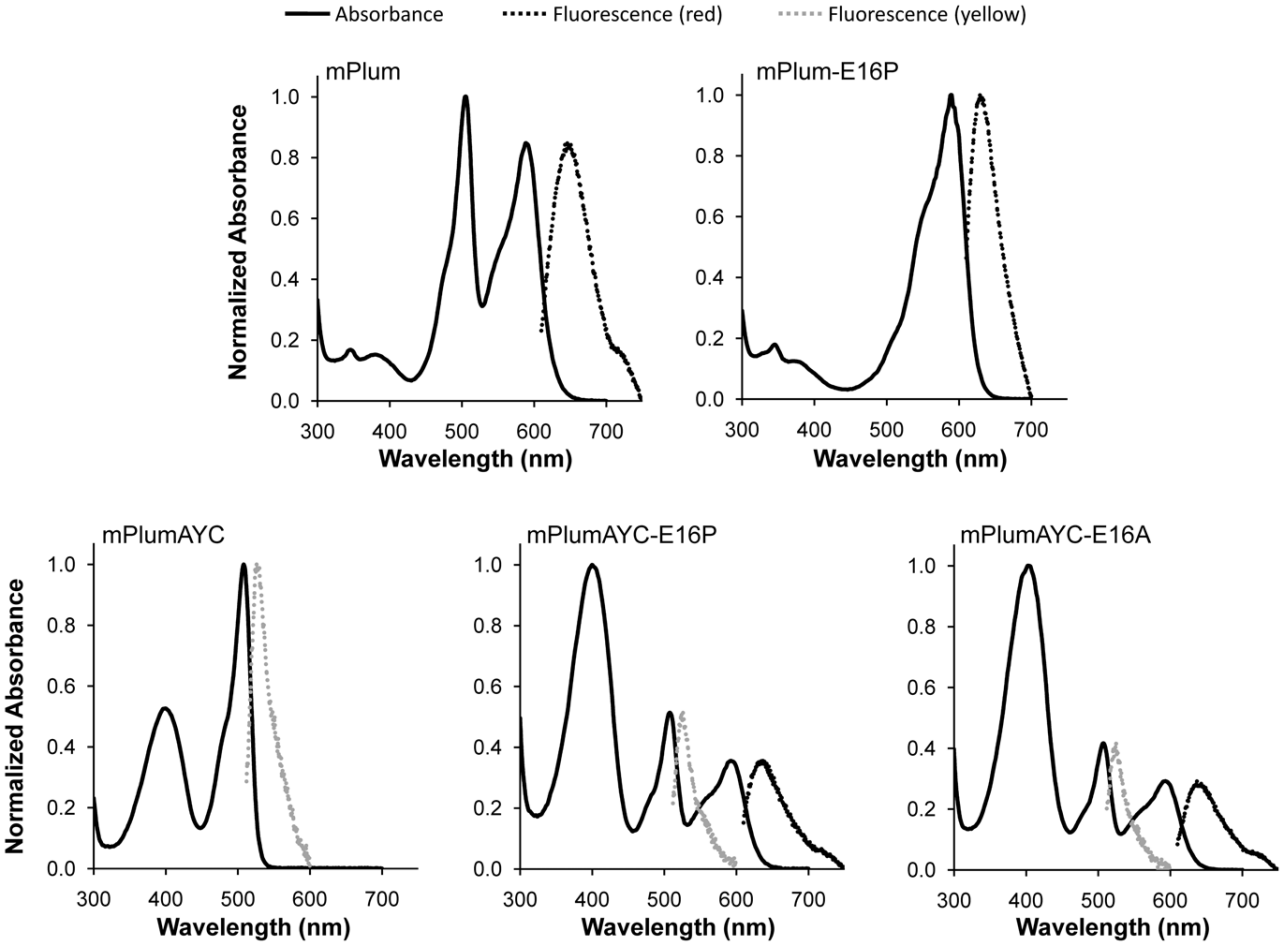
Absorption and fluorescence spectra of various RFPs. Absorption spectra (full lines) are normalized to the largest intensity absorbance peak present in each spectrum. Fluorescence emission spectra (dotted lines) are normalized to the absorbance peak in each spectrum corresponding to the excitation wavelength used to induce fluorescence. All spectra were measured at pH 7.0.

Transplanting the AYC motif into mPlum to generate mPlumAYC resulted in a mutant protein that was completely deficient in red chromophore maturation; i.e., red chromophore formation was essentially undetectable by either absorption or fluorescence spectroscopy (Fig. 2). The absorption peak corresponding to the anionic red chromophore (Fig. 1, ***10***), which is at 588 nm in the spectrum of mPlum, is absent in the spectrum of mPlumAYC. This variant instead displays peaks centered at 396 nm and 508 nm; the 396 nm peak corresponds to the neutral green chromophore (Fig. 1, ***4***), and the 508 nm peak corresponds to the anionic green chromophore (Fig. 1, ***5***). As seen in Figure S1, when the pH is increased from 7.0 to 9.5, the intensity of the 396 nm peak decreases and a concomitant increase in intensity of the 508 nm peak is observed, indicating ionization of the green chromophore. This same behavior is observed in mPlum (Fig. S1). Given that the mPlumAYC green chromophore pKa is 7.3 (Table 1, Fig. S2), peaks corresponding to both ionization states of the green chromophore should be observable at the pH value of the measurements (pH 7.0). When excited at 508 nm, mPlumAYC displayed yellow fluorescence at 527 nm with a quantum yield of 0.02, and no red fluorescence was detected with excitation at 590 nm.

**Table 1:** Properties of the RFP mPlum and its mutants.

	Mutations				Post-maturation absorbance peaks (nm)			Fluorescence emission properties: peak wavelength (nm) and quantum yield				pKa	
Protein	16	195	197	217	λ (green)^a^	λ (green)^b^	λ (red)	λ (green)	Φ_F_ (green)^c^	λ (red)	Φ_F_ (red)^c^	green	red
mPlum	E	T	I	A	391	506	588	–	–	649	0.10	N.D.	4.2
mPlum-E16P	P	T	I	A	–	509	590	–	–	630	0.14	–	4.7
mPlumAYC	E	A	Y	C	396	508	–	527	0.02	–	–	7.3	–
mPlumAYC-E16P	P	A	Y	C	395	508	594	525	0.02	637	0.04	7.4	5.4
mPlumAYC-E16A	A	A	Y	C	393	507	592	523	0.02	639	0.05	7.5	5.4

a Neutral green chromophore.

b Anionic green chromophore.

c Quantum yields for all proteins were measured at pH 7.5. mPlum exhibits measureable green-yellow fluorescence emission at pH >7.5, but at pH 7.5, it was not possible to measure a **Φ_F_** (green) value.

N.D. = not determined.

“–” indicates an absence of corresponding species and associated properties.

**Figure 3:**
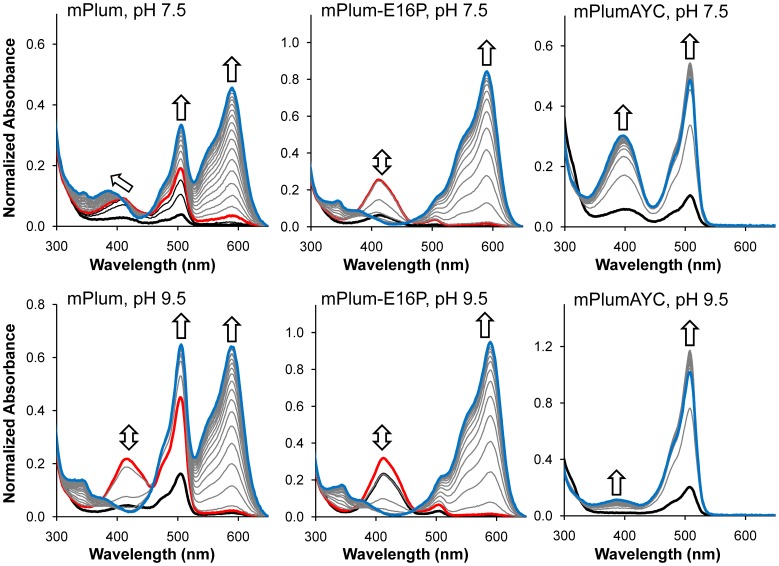
Maturation experiments. All spectra are normalized to the 280 nm absorbance peak. Heavy black and blue traces represent the beginning (t = 0 h) and end (t = 20 h) of the maturation experiment, respectively. The distance in time between each gray or black trace is 1.0 h. Arrows indicate the primary direction of peak movement during maturation. Each heavy red trace indicates the point in time when the 410 nm absorbance peak reached its maximum during the course of maturation. Black traces occur before the 410 nm peak reaches its maximum level; gray traces occur after the maximum.

**Figure 4:**
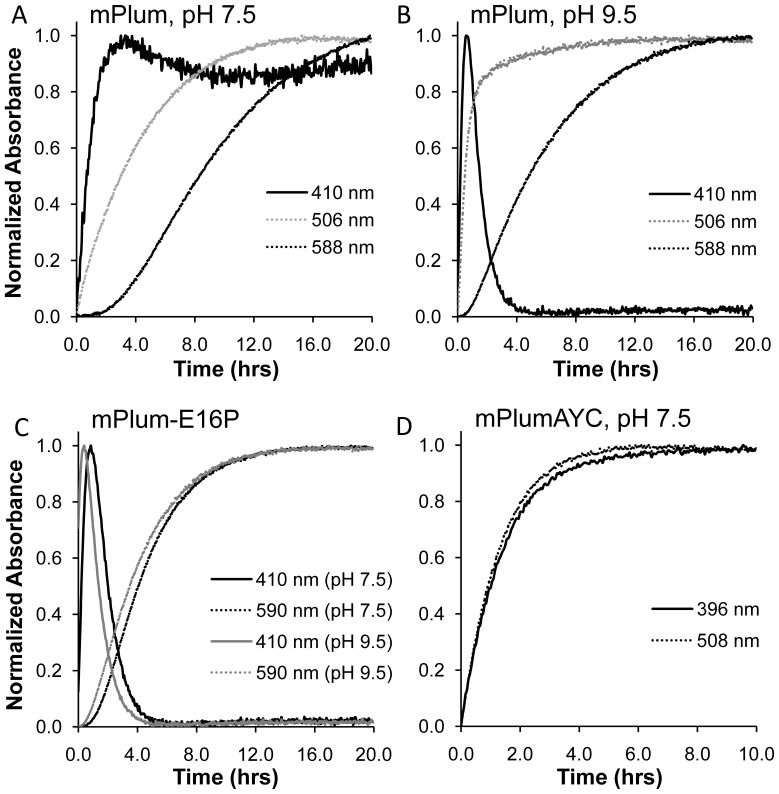
Maturation kinetics plots. All spectral data is normalized to the maximum peak intensity observed over the course of maturation for each wavelength depicted. Suppression of spectral interference involving the 410 nm absorbance peak is illustrated for mPlum when maturation is tracked at higher pH (A and B). A shift to faster red chromophore maturation half-time and faster arrival at the 410 nm peak maximum occurs when tracking maturation at pH 9.5 in mPlum-E16P (C). This shift to shorter half-times can be seen in mPlum as well for pH 7.5 (A) versus pH 9.5 (B). In mPlumAYC (D), green chromophore maturation half-time is equivalent when tracking both the neutral green chromophore (396 nm) and the anionic green chromophore (508 nm). This result indicates that green chromophore ionization and maturation occur on much different timescales.

To examine the effect of the AYC motif on chromophore maturation, we performed maturation experiments. RFPs were expressed anaerobically in airtight culture tubes and purified rapidly at 4°C in deoxygenated solutions to obtain protein samples that contained few or no fully mature chromophores. Absorption scans were then done to follow the maturation process at 28°C. Chromophore maturation of mPlum at pH 7.5 (Figs. 3 and 4A) demonstrates an increase in absorbance as a function of time for the anionic green (506 nm) and red (588 nm) chromophore peaks. Additionally, a peak at 410 nm appears to shift to 391 nm during the maturation process at pH 7.5. The peak at 410 nm corresponds to the blue intermediate species [7], [10] (Fig. 1, ***7***), and the peak at 391 nm corresponds to the neutral green chromophore (Fig. 1, ***4***). To remove spectral overlap of these two peaks, we performed the same maturation experiments at pH 9.5 (Fig. 3). At this pH, the neutral green chromophore is converted to the anionic green chromophore via deprotonation, thereby decreasing the peak intensity at 391 nm and leaving the 410 nm peak unaffected. mPlum maturation at pH 9.5 (Figs. 3 and 4B) shows an increase in absorbance as a function of time for the anionic green (508 nm) and red (588 nm) chromophore peaks. Additionally, a peak at 410 nm increases in intensity and then disappears. This result confirms that the blue intermediate is formed transiently during maturation, as expected [6], [7], [22].

For mPlumAYC, increases in absorbance are observed during maturation at pH 7.5 for the neutral (396 nm) and anionic (508 nm) green chromophore peaks. At pH 9.5, the 396 nm peak disappears due to ionization of the neutral green chromophore, which would allow a 410 nm peak to be observed, if present. For mPlumAYC, however, no such peak is observed (Fig. 3), indicating that the intermediate blue species does not form and that the second oxidation reaction (Fig. 1, ***6*** to ***7***) does not occur. No evidence for the presence of the red chromophore was observed in mPlumAYC as indicated by the absence of any absorption peak at >550 nm (Fig. 2). Thus, introduction of the AYC motif into mPlum prevents red chromophore maturation by inhibiting the second oxidation reaction, which results in a dim yellow fluorescent protein containing only the green chromophore.

### Recovery of Red Chromophore Maturation through Rational Design

Chromophore maturation in mPlum results in a mixture of protein molecules containing either the red chromophore (λ = 588 nm) or the green chromophore (peaks at 391 nm and 506 nm) [12] (Fig. 2). This is not the case for mCherry, a fast and efficient maturing RFP that matures almost exclusively to the red chromophore with a single peak centered at 586 nm (Fig. S1) [15]. We hypothesized that Glu16 plays an important role in mPlum’s inefficient red chromophore maturation compared to mCherry. Our hypothesis is based on the fact that Glu16 H-bonds to the chromophore acylimine oxygen atom [21]. We postulated that this H-bonding interaction could restrict the conformational freedom of the peptide backbone, which may be required for the second oxidation reaction generating the acylimine group to take place. Previously, researchers suggested that a *cis*-*trans* isomerization of the peptide backbone between residues 65 and 66 was necessary for this second oxidation reaction to occur [5], [23]. However, such an isomerization would result in a red chromophore with an acylimine oxygen atom pointing up away from catalytic residue Glu215; this has never been observed [24]. During the second oxidation reaction, the C-alpha carbon of residue 66 is transformed from sp^3^-hybrization to sp^2^-hybridization, changing its geometry from tetrahedral to trigonal planar. This process clearly requires a conformational change in the peptide backbone of residue 66. An H-bonding interaction between Glu16 and the carbonyl oxygen of residue 65 would very likely decrease conformational flexibility around this bond.

An additional observation that lends support to our hypothesis comes from the absorption spectrum of mRojoA [18], an mCherry mutant that contains the AYC motif as well as a water-mediated H-bond between the side chain of Thr16 and the chromophore acylimine oxygen. Absorption spectra for mRojoA demonstrate that as the pH is increased from 7.0 to 9.5, a peak at 517 nm is revealed, indicating that mRojoA expresses as a mixture of green and red chromophore-containing proteins (Fig. S1). mCherry, which contains a non-H-bonding valine at position 16, does not exhibit this behavior (Fig. S1), indicating that purified preparations of this protein are essentially homogeneous in their maturation to the red chromophore.

Based on these observations, we hypothesized that replacement of Glu16 with residues that cannot donate H-bonds to the acylimine oxygen would improve maturation to the red chromophore in maturation-deficient mPlumAYC. To test this hypothesis, we performed mutagenesis at position 16 in mPlumAYC followed by fluorescence detection of mutants that displayed emission at wavelengths >550 nm. A total of 11 different amino acids including glutamic acid were tested at position 16; these included mutations to polar, hydrophobic, aromatic, acidic, and basic residues (Fig. S3). The E16D and E16T mutations did not recover much red fluorescence in mPlumAYC, suggesting that side chains that can participate in H-bonding with the acylimine oxygen are detrimental to the second oxidation reaction leading to the red chromophore. The polar residues tyrosine and arginine also did not recover red fluorescence.

Mutation to either an alanine or a proline resulted in the highest recovery of red fluorescence intensity. Some recovery was seen for other non-polar mutations (E16G, E16V, E16I and E16F), but the average integrated red fluorescence signal for these mutants was less than that seen for E16P and E16A (Fig. S3). Decreased fluorescence intensity may be due to lower protein expression levels or impaired fluorescence. However, since cellular densities were controlled, discrepancies in integrated fluorescence intensity are unlikely to result from well-to-well differences in cellular growth levels. Thus, our mutagenesis study revealed that small non-polar residues improve red chromophore maturation efficiency, possibly by allowing for conformational flexibility during oxidation of the peptide backbone that generates the acylimine. Additionally, the presence of side chains smaller than glutamic acid at position 16 may increase molecular oxygen accessibility to the peptide backbone, thereby improving the initiation efficiency of this oxidation reaction.

The two mutants that displayed the highest red fluorescence intensity, mPlumAYC-E16A and mPlumAYC-E16P, were further characterized. Their absorption spectra displayed three peaks centered at ∼390 nm (neutral green chromophore), ∼510 nm (anionic green chromophore), and ∼590 nm (red chromophore), similar to what is observed for mPlum (Fig. 2). However, unlike mPlum, the highest intensity peaks for both of these mutants in the pH 7.0–9.5 range are the ∼390 nm and ∼510 nm peaks (Figs. 2 and S1), suggesting that the majority of RFP molecules contain a green chromophore instead of a red chromophore. The red/green chromophore ratios are reported in Table 2 as ratios of absorbance at 590 nm and 510 nm (A_590_/A_510_). These ratios were calculated using absorbance values measured at pH 9.5 in order to deprotonate all green chromophores present in solution, converting the entirety of green chromophore-related absorbance to a single peak around 510 nm. The red/green chromophore ratio for mPlum is close to unity, whereas it is approximately 0.15 for mPlumAYC-E16A and mPlumAYC-E16P. Since the extinction coefficients of the red and green chromophores in FPs do not typically differ by orders of magnitude, this change in red/green ratio is significant. When excited at 510 nm, mPlumAYC-E16A and mPlumAYC-E16P display yellow fluorescence at 523 nm and 525 nm, respectively, with Φ_F_ = 0.02. These proteins thus contain a significant proportion of molecules that contain a yellow-emitting green chromophore, similar to mPlumAYC.

**Table 2:** Maturation data.

	Maturation half-time (green)^a^ (h)		Maturation half-time (red) (h)		Time of 410 nm peak maximum (h)	A_590_/A_510_ in mature protein	NZ-O2 distance
Protein	pH 7.5	pH 9.5	pH 7.5	pH 9.5	pH 9.5	pH 9.5	(Å)^b^
mPlum	2.8±0.2	0.47±0.04	7.3±0.1	3.90±0.09	0.62±0.03	0.9	5.2
mPlum-E16P	3.12±0.06	2.18±0.06	3.77±0.03	3.12±0.08	0.38±0.03	3.7	4.2
mPlumAYC	0.89±0.04	0.79±0.01	–c	–c	–c	–c	6.3
mPlumAYC-E16P	1.43±0.03	1.24±0.04	5.4±0.3	5.6±0.4	2.8±0.1	0.14	–
mPlumAYC-E16A	1.83±0.07	1.20±0.05	5.9±0.2	4.6±0.1	3.8±0.4	0.15	6.2

a Maturation half-times are for the anionic green chromophore that absorbs at ∼510 nm.

b Crystallographic NZ-O2 distances are presented for the major conformer of Lys70 in each structure.

c Does not mature to the 410 nm absorbing blue intermediate or to the anionic red chromophore.

mPlumAYC-E16A and mPlumAYC-E16P also mature to molecules containing red chromophores that emit at 639 nm and 637 nm, respectively (Table 1). Interestingly, these mutants emit at wavelengths 10–12 nm shorter than mPlum (λ_em_ = 649 nm). This is presumably because they have lost the dynamic Stokes shift resulting from the H-bonding interaction between the chromophore acylimine and Glu16. Other mPlum mutants with non-polar amino acids at position 16 have been shown to emit at 615–626 nm [20], [21]. Since our mPlumAYC-E16A and mPlumAYC-E16P mutants emit at 637–639 nm, we propose that their 11–24 nm longer emission wavelengths result from the red-shifting effect of the π-stacking interaction incorporated through the AYC motif. Although the E16P and E16A mutations in mPlumAYC partially restore red chromophore maturation, the red fluorescence quantum yields for these mutants (Φ_F_ = 0.04 and 0.05, respectively) are about half that of mPlum (Table 1).

Maturation kinetics experiments on mPlumAYC-E16A and mPlumAYC-E16P revealed that both variants display a time-dependent increase in the peaks corresponding to the green (∼390 and ∼510 nm) and red (∼590 nm) chromophores at pH 7.5 (Fig. S4). As observed for mPlum, the ∼390 nm peak overlaps with the 410 nm peak of the blue intermediate species. To remove this spectral overlap, maturation experiments were also performed at pH 9.5 (Fig. S4). At this higher pH, we observe a peak at 410 nm that increases in intensity and then disappears, suggesting that the blue intermediate species has been consumed to form the red chromophore, as seen for mPlum (Fig. 3). Thus, loss of the H-bonding interaction between residue 16 and the acylimine oxygen appears to partially restore oxidation of the backbone with subsequent formation of the intermediate blue species (Fig. 1, ***7***).

### A Fast and Efficient Red Chromophore Maturing mPlum Mutant

A disadvantage of mPlum for imaging experiments is that preparations of this protein result in a mixture of molecules that contain either red or green chromophores [12], as observed in Figure 2. This is an undesirable property for multicolor imaging applications. To assess whether removal of the H-bonding interaction between Glu16 and the chromophore would result in improved maturation to the red chromophore in mPlum, we prepared the point mutant mPlum-E16P and measured its absorption spectrum. This single amino acid change resulted in an RFP that matures almost exclusively to the red chromophore (Fig. 2). The 506 nm anionic green chromophore peak at pH 7.0 was ablated, and only one major peak located at 590 nm is observed. When the pH is increased to 9.5 (Fig. S1), a small but observable peak appears at 509 nm, corresponding to the anionic green chromophore. These results indicate that maturation to the red chromophore in mPlum-E16P, although dramatically improved, is not complete. Nevertheless, the absorbance ratio A_590_/A_510_ is four times that of mPlum (Table 1), indicating that red chromophore-containing molecules constitute the majority of the RFP population in solution. Maturation kinetics experiments of mPlum-E16P at both pH 7.5 and 9.5 show a clearly observable 410 nm peak corresponding to the intermediate blue species (Figs. 3 and 4C). The blue species is formed rapidly and is consumed to produce the red chromophore. No peak for the neutral green chromophore is present to overlap with the 410 nm peak, again suggesting that little green chromophore is present. Therefore, the E16P mutation improved red chromophore maturation efficiency in mPlum, similar to its effect in mPlumAYC.

mPlum-E16P emits red fluorescence at 630 nm, which is almost 20 nm blue-shifted relative to mPlum. This indicates that the dynamic Stokes shift responsible for the large red shift in mPlum is lost when Glu16 is replaced by a proline. Interestingly, this 630 nm emission wavelength is identical to that of mPlum-E16Q, which can still form an H-bond with the chromophore acylimine group [21]. Moreover, the emission wavelength of mPlum-E16P is 12 nm longer than that of the E16L mutant, which cannot form such an interaction [21]. Notably, the quantum yield of mPlum-E16P is 40% higher than the quantum yield of mPlum (Table 1) and its extinction coefficient of 29,350±2,000 M^–1^ cm^–1^ is similar to that of mPlum (22,000 M^–1^ cm^–1^) [25], resulting in a brighter RFP. Another characteristic of mPlum-E16P is that its excitation at 509 nm does not result in detectable yellow fluorescence. Hence, removal of the Glu16-acylimine H-bond largely improved red chromophore maturation in mPlum.

### Chromophore Maturation Mechanism

**Figure 5:**
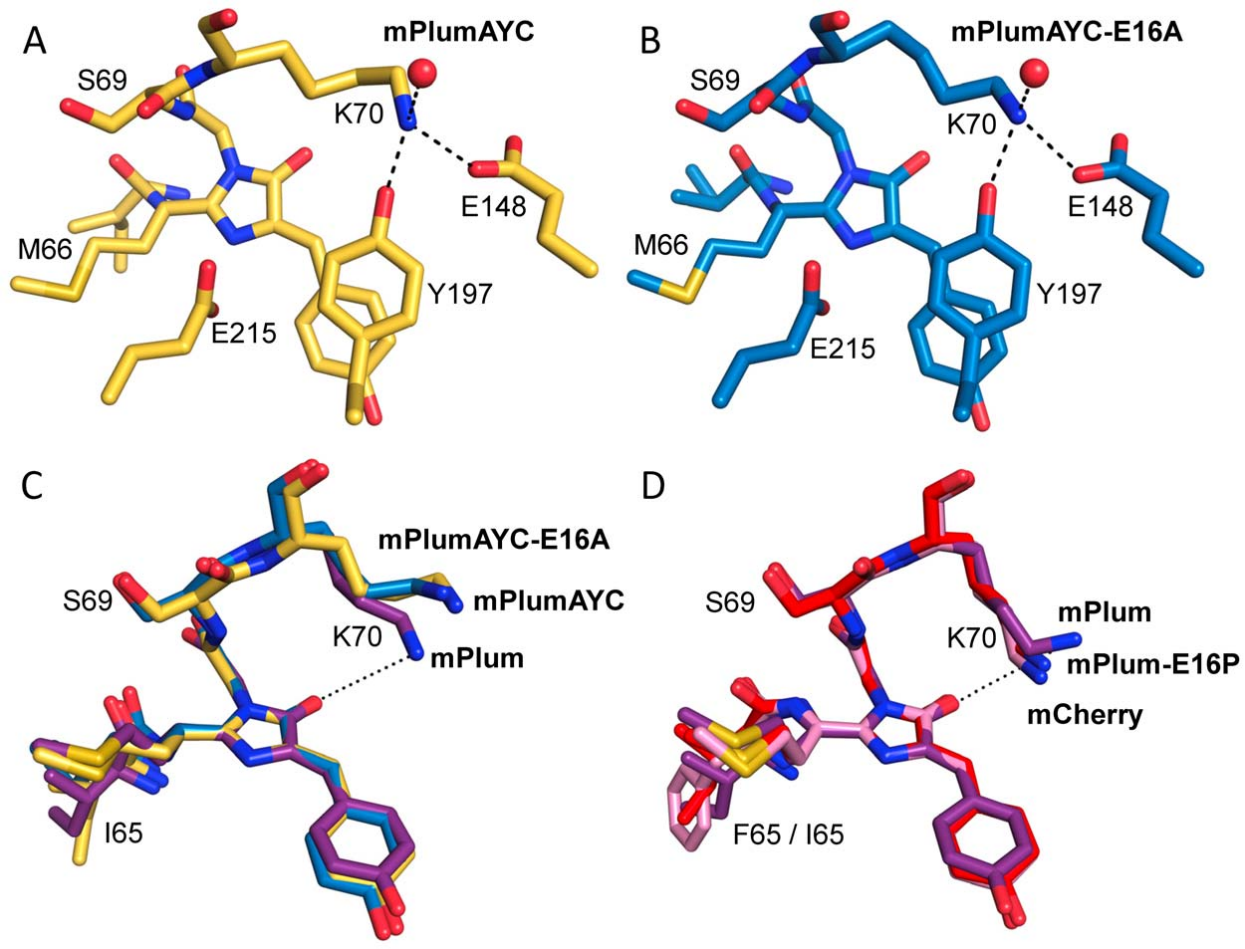
Crystal structures. (A and B) Introduction of the AYC motif results in π-stacking interactions between the chromophore and Tyr197 in both mPlumAYC (A) and mPlumAYC-E16A (B). H-bonding interactions with Lys70 are illustrated with dashed lines. These interactions combine to sequester the terminal amino group of Lys70 away from the chromophore. (C and D) Comparisons of Lys70-to-chromophore distance are illustrated between mPlum (purple), mPlumAYC (yellow), and mPlumAYC-E16A (blue) (C), as well as between mCherry (pink), mPlum (purple), and mPlum-E16P (red) (D). A dotted line connects the NZ atom of Lys70 in mPlum to the O2 atom of the mPlum green (C) or red (D) chromophore. Note that Lys70 in mPlum (PDB code 2QLG [21]) adopts a slightly different conformation in the green versus red chromophore contexts. All proteins were aligned by the atoms of their imidazolinone ring.

To better understand the roles of the AYC motif and position 16 residue identity during chromophore maturation, we performed maturation kinetics experiments for all the mPlum-derived RFPs described above (mPlum, mPlum-E16P, mPlumAYC, mPlumAYC-E16P, and mPlumAYC-E16A). These experiments were performed at pH 7.5 to approximate physiological pH and at pH 9.5 to avoid spectral overlap of the ∼390 nm and 410 nm absorption peaks. Maturation half-times for the anionic green and red chromophores (Fig. 1, ***5*** and ***10***) were measured, as well as the time to reach maximum 410 nm absorbance. Maturation half-times for the formation of the neutral green chromophore (Fig. 1, ***4***) were not measured for two reasons. First, the ∼390 nm peak corresponding to this species overlaps with that of the 410 nm absorbing blue intermediate, introducing measurement errors. Second, maturation data from mPlumAYC, which matures exclusively to the green chromophore, revealed that maturation half-times for the neutral and anionic green chromophores were identical at the pH of measurement (Fig. 4D). This result indicates that ionization of the green chromophore occurs on a much faster timescale than chromophore formation. Thus, the maturation half-time of the anionic green chromophore is sufficient to accurately reflect the maturation half-time of the dehydration step leading to the neutral green chromophore (Fig. 1, ***3*** to***4***).

For the RFPs studied here, maturation half-times of both green and red chromophores were faster at pH 9.5 than at pH 7.5, except for mPlumAYC-E16P (Table 2). In this protein, red chromophore formation half-times were not significantly different at these two pHs. A shortening of maturation half-times in response to increased pH suggests that a base may be involved in the rate-limiting step on both the green and red chromophore maturation pathways. This assessment is supported by previous studies in which dehydration along both maturation pathways was shown to be rate limiting [7], [11]. Since dehydration reactions in the chromophore maturation mechanism (Fig. 1) require proton abstraction to eliminate hydroxide, it is expected that a higher pH would accelerate these reactions. Interestingly, the greatest acceleration in maturation as a function of pH occurs for mPlum (Table 2 and Fig. 4A,B). For green chromophore maturation, the half-time decreases from 2.8 h at pH 7.5 to 0.47 h at pH 9.5, whereas for the red chromophore, maturation half-time decreases from 7.3 to 3.9 h. It is unclear why only mPlum would display such a large acceleration of chromophore maturation, but ionization of the Glu16 residue at higher pH could be a factor.

For all the RFPs studied here, the first peak that appears during chromophore maturation is the 410 nm blue species peak, followed by the green chromophore ∼510 nm peak, and finally the red chromophore ∼590 nm peak (Figs. 3, 4, and S4). Formation of the blue intermediate before the red chromophore is expected. Formation of this blue species by the second oxidation reaction has been demonstrated to shunt intermediates down the red chromophore-forming pathway [7]. Studies in other RFPs have shown similar behaviors [6], [8]. The times at which the 410 nm peak reaches maximum absorbance are reported in Table 2 for all RFPs studied here. After this time point, the production rate of the blue species falls behind the consumption rate, and this species is continually transformed into red chromophore. Except in mPlum-E16P, the 410 nm peak maximum is reached after the green chromophore maturation half-time. This observation indicates that the blue intermediate species is still forming when half of the total green chromophores in solution have been produced. Importantly, the relative rates for the dehydration step leading to green chromophore formation (Fig. 1, ***3*** to ***4***) and the oxidation step leading to the acylimine-containing blue species (Fig. 1, ***6*** to ***7***) should determine the final ratio of red to green chromophore-containing molecules. Thus, a kinetic effect at the branch point of the mechanism (Fig. 1, boxed) should determine the efficiency of RFP chromophore maturation. Consequently, if the half-time of green chromophore maturation is shorter than the time of peak maximum for the blue species, a significant proportion of molecules should contain green chromophores. This is indeed what is observed for mPlum, mPlumAYC-E16A, and mPlumAYC-E16P, which have red/green chromophore ratios less than 1.0 (Table 2). On the other hand, if formation of the blue species is faster than the dehydration step, the final ratio of red/green chromophores should increase. This is what we observe for our mPlum variant with the most efficient red chromophore maturation, mPlum-E16P, whose 410 nm peak maximum is reached 1.8 h before its green chromophore maturation half-time (Table 2). Thus, in mPlum-E16P, before half of the total green chromophore is formed, the majority of flux through the blue species has already occurred via consumption along the pathway to yield red chromophore. As a result, at the completion of the maturation process, this protein displays the highest red/green ratio of all mPlum mutants described here (A_590_/A_510_ = 3.7).

**Figure 6:**
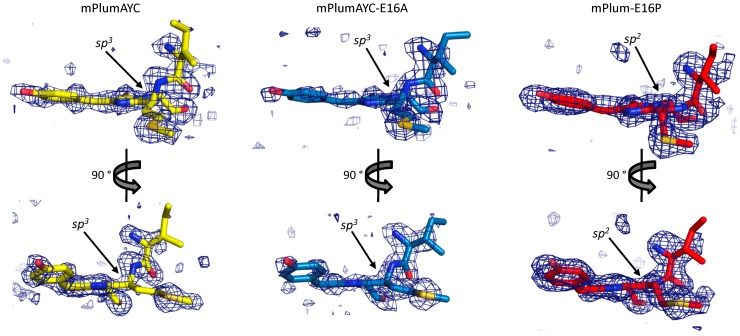
Electron density maps. Omit maps contoured at 3σ were constructed for the chromophore and surrounding main chain atoms in mPlumAYC, mPlumAYC-E16A, and mPlum-E16P. Arrows indicate the sp^2^ or sp^3^-hybridized alpha carbon atom of Met66 observable in each chromophore.

In summary, our data support the notion that to obtain RFPs displaying more efficient red chromophore maturation, the blue species formation rate must exceed the green chromophore formation rate. The overall red chromophore formation rate need not be increased outright, since the half-time of red chromophore maturation is always longer than the half-time of green chromophore maturation (Table 2) [6], [7]. Presumably, the E16A and E16P mutations speed up the second oxidation step and possibly slow down the dehydration step leading to green chromophore formation. Alternatively, the competition for intermediate substrates (Fig. 1, ***3*** and ***6***) becomes more pronounced when the second oxidation step is made more efficient, which then impedes dehydration. For example, Table 2 shows that the maximum absorbance of the 410 nm peak is reached ∼0.2 h faster during mPlum-E16P maturation than during maturation of mPlum. Simultaneously, the maturation half-time of the green chromophore is lengthened in mPlum-E16P relative to mPlum at both pH 7.5 and pH 9.5. Longer green chromophore maturation half-times are also observable for the mPlumAYC-E16P and mPlumAYC-E16A mutants relative to mPlumAYC (Table 2).

Lastly, the AYC motif affects maturation by slowing down formation of the blue species, and in the case of mPlumAYC, by completely inhibiting it. As seen in Table 2, the time of 410 nm peak maximum is much longer for the AYC motif-containing mPlum mutants than for mPlum and mPlum-E16P, which do not contain this motif. At the same time, the AYC mutations appear to speed up dehydration leading to the green chromophore. For example, at pH 7.5, green chromophore maturation half-time is ∼2 h shorter in mPlumAYC than in mPlum and is ∼1.7 h shorter in mPlumAYC-E16P than in mPlum-E16P. At pH 9.5, green chromophore formation remains faster for mPlumAYC-E16P compared to mPlum-E16P. Notably, the green chromophore formation rate for mPlum exceeds that for mPlumAYC at pH 9.5, but this may be related to interfering effects from ionization of Glu16 at higher pH. On balance, the AYC motif appears to favor the dehydration reaction leading to formation of the green chromophore (Fig. 1, ***3*** to ***4***) over the second oxidation reaction (Fig. 1, ***6*** to ***7***) that eventually leads to red chromophore formation.

### Crystallographic Support of Maturation Studies

The various mPlum mutants described here constitute a useful set of proteins for studying the structure/function relationships involved in RFP chromophore maturation. Thus, to help elucidate the mechanisms underlying the observed effects of our mutations on chromophore maturation, we solved the crystal structures of mPlum-E16P, mPlumAYC, and mPlumAYC-E16A. Data collection and refinement statistics are reported in Table S1. The structure of mPlumAYC demonstrates that the intended π-stacking interaction between Tyr197 and the chromophore was successfully introduced (Fig. 5A). The centroid-to-centroid distance between the Tyr197 phenol ring and the chromophore phenol ring was 3.0 Å and the interplanar angle between these rings was 12.3°. In the mPlumAYC-E16A structure (Fig. 5B), the centroid-to-centroid distances were 4.1 Å and 4.0 Å and the interplanar angles were 11.6° and 11.1° for chains A and B, respectively. For comparison, the π-stacking interaction of the yellow fluorescent protein citrine has a centroid-to-centroid distance of 3.6 Å and an interplanar angle of 6.1° [26]. In mRojoA, an mCherry mutant equipped with the AYC motif [18], centroid-to-centroid distances are tightly clustered in a range of 3.8–3.9 Å for all subunits in the crystal, and the interplanar angles span 4.2°–12.8°. Thus, our crystallographic data demonstrate that introduction of the AYC motif in mPlum resulted in successful creation of the desired π-stacking interaction with the chromophore.

The crystal structures of mPlumAYC and mPlumAYC-E16A did not contain appreciable omit map density (contoured at 3σ) corresponding to the presence of an oxidized peptide backbone with sp^2^-hybridized C-alpha carbon at residue 66, which is indicative of red chromophore formation. However, these structures did show well-resolved tetrahedral sp^3^-hybridized C-alpha atoms at position 66 (Fig. 6), indicating green chromophores. This observation is consistent with the weaker intensity observed for the red chromophore absorption peak (∼590 nm) of mPlumAYC-E16A and for the absence of such a peak for mPlumAYC (Fig. 2). This weaker or absent peak corresponds to a concentration of red chromophore-containing molecules that is substantially lower or absent. In turn, this smaller population of red chromophore molecules in solution contributes to a smaller population of these molecules in the crystal and consequently an absence of appreciable electron density. Conversely, the structure of mPlum-E16P presents no appreciable omit map electron density for the green chromophore when contoured at 3σ. Only density corresponding to a planar sp^2^-hybridized C-alpha carbon at residue 66 (Fig. 6), indicating the red chromophore, is apparent. Notably, this correlates with the near complete red chromophore maturation observed for mPlum-E16P. During analysis of the electron density in all three structures, occupancy refinement starting with 50% of both red and green chromophores terminated by driving the appropriate green or red population to 0% occupancy.

When comparing the crystal structures of mPlum (PDB code 2QLG) [21], mPlum-E16P, mPlumAYC, and mPlumAYC-E16A, we observed strong overall similarity for residues surrounding the chromophore, including the catalytic residues Arg96 and Glu215. On the acylimine side of the chromophore, the Pro16 residue in mPlum-E16P appears to slightly distort the secondary structure of the first β-strand in the protein, but does not cause any major structural changes. The only appreciable difference we could find among these structures was in the crystallographic side chain conformation of Lys70 (Fig. 5C, D). In mPlum-E16P, the Lys70 side chain adopts a conformation similar to that found in the fast and efficient maturing RFP mCherry [4]. However, in mPlum, the red chromophore-adjacent Lys70 residue adopts a slightly different conformation, causing the NZ atom to retract away from the chromophore by 1.4 Å with respect to its position in mPlum-E16P (Fig. 5D). In mPlumAYC and mPlumAYC-E16A, the presence of Tyr197 blocks the space where the NZ atom of the Lys70 side chain in mPlum-E16P and mCherry would sit. In these AYC motif-containing proteins, the side chain of Lys70 forms three H-bonding interactions: one with the side chain of Glu148, another with the side chain of Tyr197, and a third with a water molecule (Fig. 5A, B). However, the side chain of Lys70 in mPlum and mPlum-E16P only forms two H-bonding interactions: one with the side chain of Glu148 and another with a water molecule. The AYC motif therefore appears to lock the side-chain amine of Lys70 into a position over 6 Å away from the chromophore (Table 2). In contrast, the side-chain amino groups of Lys70 in mPlum-E16P and mPlum are able to sit about 4 Å and 5 Å away (Table 2), respectively, from the nearest red chromophore heavy atom (O2, the imidazolinone oxygen). Thus, we propose that the AYC motif slows down (mPlumAYC-E16A and mPlumAYC-E16P) or completely hinders (mPlumAYC) the second oxidation reaction during chromophore maturation by pushing Lys70 away from the chromophore and locking it into a more distal conformation through an additional H-bond. Thus, sequestration of the Lys70 side chain away from the chromophore results in the observed maturation deficiency.

In support of the above statement, we see a general trend toward less efficient red chromophore maturation for longer NZ-to-chromophore distances in our mPlum mutants. Specifically, we observe a negative correlation between the Lys70 NZ-to-chromophore distance and the red/green ratio (Table 2). Most DsRed-derived RFPs have a conserved lysine at position 70, and it was shown that the K70M mutation in DsRed suppresses red chromophore formation completely [3], [13]. Recently, Lys70 was proposed to be important for the formation of the intermediate blue species through an electrostatic interaction with the chromophore’s imidazolinone oxygen atom [10]. Our observation that proximity to the chromophore of the Lys70 side chain correlates with RFPs that mature more efficiently agrees with this proposal.

### Maturation Promoting Mutations

Because FPs need to fold before any of the chemical transformations required to produce the chromophore can occur [27], [28], chromophore maturation is the rate-limiting step for FPs to become fluorescent. For most imaging experiments, RFPs that mature quickly to the red chromophore are preferred. Commonly-used FPs mature with half-times of 40 min to 2 h at 37°C [29]. To eliminate background signal, complete maturation to the red chromophore is also desirable. As a result, much effort has been devoted to the optimization of red chromophore maturation in RFPs [15], [16], [30].

Mutations that improve red chromophore maturation in RFPs have been discovered primarily through screening of large mutant libraries obtained through random mutagenesis. Much work has focused on improving DsRed maturation, resulting in a number of mutants displaying accelerated maturation and decreased green chromophore formation [16], [30], [31], [32]. Positions in the sequence of DsRed that are known to affect chromophore maturation include residues 42, 66, 70, 71, 83, 105, 163, and 217. DsRed mutations N42Q [16], V105A [31], Q66M [3], [32], and M163Q [32] have been shown to improve maturation by accelerating red chromophore formation or decreasing green chromophore formation. On the contrary, mutations V71M [31], K70M [13], and K83R [33] have been shown to inhibit red chromophore formation and result in green fluorescent proteins. Of the residues involved in the AYC motif, position 217 has been shown to accelerate red chromophore maturation when mutated to alanine [16].

In this study, mutations that hinder or improve red chromophore maturation in DsRed-derived RFPs were rationally designed. We converted the red fluorescent protein mPlum into a strictly yellow-emitting FP by introducing three mutations around the chromophore (T195A, I197Y, and A217C). Then, we used rational design to successfully reintroduce red chromophore formation into mPlumAYC and to drastically improve red chromophore maturation efficiency in mPlum. The rationally designed mutations that resulted in the greatest improvement of chromophore maturation efficiency were E16A and E16P.

One other example of the conversion of green chromophore-containing FPs into RFPs via mutagenesis has been reported. In 2008, Mishin et al. used random mutagenesis and high-throughput screening to convert *Aequorea victoria* GFP into a mutant that emits at 585 nm [34]. The authors proposed that the red fluorescence-emitting GFP mutant undergoes a dehydration reaction at Ser65 of the chromogenic tripeptide (Ser65-Tyr66-Gly67), which results in a dehydroalanine residue that tautomerizes to an acylimine group. The presence of this acylimine extends the conjugation of the chromophore, resulting in red fluorescence at 585 nm. Unlike this prior study, our results represent the first instance in which a green chromophore-containing FP was converted to a red chromophore-containing FP by rational design. Moreover, this conversion was accomplished without reverting any of the mutations causing the maturation deficiency and without altering the red chromophore’s covalent structure.

### Conclusions

In this study, we converted a yellow fluorescent mutant of mPlum into a red-emitting RFP using principles of rational design. Based on our observations, we propose two structural features that are important for efficient red chromophore formation in DsRed-derived RFPs. The first is the presence of a lysine residue at position 70 that is able to interact directly with the chromophore. Such an interaction has already been shown to be important for red chromophore formation [3], [10]. To interact efficiently with the chromophore, this lysine must be within a certain distance (≤5 Å) and must not be locked in a conformation that sequesters the side chain NZ atom away from the chromophore. The second structural feature is an absence of non-bonding interactions limiting the conformational flexibility at the peptide bond that is oxidized to form an acylimine during red chromophore formation. Since the geometry of the C-alpha atom in the first amino acid of the chromogenic tripeptide (residue 66 for mPlum) must change from tetrahedral to trigonal planar during oxidation, flexibility is needed for this process to occur efficiently. Our data, as well as structural features for known acylimine-forming FPs, support this proposition. Satisfying or improving these structural features in other maturation-deficient RFPs may result in RFPs with faster and more complete maturation to the red chromophore.

## Materials and Methods

### Materials

All reagents used were of the highest available purity. Restriction enzymes and DNA-modifying enzymes were from New England Biolabs. Synthetic oligonucleotides were obtained from Integrated DNA Technologies, and Ni-NTA agarose resin was obtained from Qiagen. CelLytic B buffer and lysozyme were purchased from Sigma-Aldrich. All aqueous solutions were prepared using water purified with a Millipore BioCell system.

### Mutagenesis

The mPlum gene was PCR-amplified from plasmid mPlum-pBAD (provided by R.Y. Tsien, UCSD) and subcloned into pET11-a (Novagen) via *Nde*I/*Bam*HI. The plasmid was then transformed into *Escherichia coli* XL-1 Blue. The entire *Nde*I/*Bam*HI fragments, including the whole coding region, were verified by DNA sequencing. All mutations were introduced into the mPlum gene by overlap extension mutagenesis [35] using VentR DNA polymerase (NEB). Briefly, external primers were used in combination with sets of complementary pairs of oligonucleotides containing the desired mutations in individual PCR reactions. The resulting overlapping fragments were gel-purified (Qiagen) and recombined by overlap extension PCR. The resulting amplicons were digested with *Nde*I/*Bam*HI, gel-purified, and ligated into pET11-a expression vector with T4 ligase.

### Characterization of mPlum Mutants

The plasmids prepared as described above were transformed into chemically competent *E. coli* BL21-Gold(DE3) cells (Stratagene). Colonies were picked into individual wells of Nunc V96 MicroWell polypropylene plates containing 200 µL of medium (LB with 100 µg/mL ampicillin supplemented with 10% glycerol). The plates were covered with a sterile Breathe-Easy gas permeable sealing membrane (Sigma) and incubated overnight at 37°C with shaking. After incubation, these mother plates were used to inoculate sterile Nunc V96 MicroWell polypropylene plates (daughter plates) containing 300 µL of Overnight Express Instant Terrific Broth media (Novagen) supplemented with 100 µg/mL ampicillin per well. Daughter plates were sealed with breathable membranes and incubated overnight (37°C, 250 rpm shaking). After incubation, the cells were harvested by centrifugation and the cell pellets were washed twice with phosphate buffered saline (PBS). Washed cell pellets were then incubated at 4°C for 72 h to allow chromophore maturation. These pellets were resuspended in PBS and transferred to a Fluotrac 96-well plate (Greiner Bio-One) for screening. Fluorescence was measured with a Tecan Safire2 plate reader. Emission spectra (λ_ex_ = 570 nm) were measured from 590 nm to 700 nm.

### Protein Expression and Purification

Protein was expressed in 1.0 L cultures by transformation of a pET11-a vector containing the gene of interest into *E. coli* BL21-Gold(DE3) and purified by Ni-NTA affinity chromatography according to the manufacturer’s protocol. Column elutions were desalted by gel filtration using a Superdex 75 10/300 GL Tricorn resin column (GE Healthcare) into a final buffer solution of 50 mM phosphate buffer, pH 7.5, and 150 mM NaCl.

### Spectroscopic Characterization

All absorption and emission spectra were recorded with a Tecan Safire2 plate reader in Greiner UV-Star 96-well plates. Proteins purified as described above were quantified using the alkali denaturation method [33]. Briefly, RFPs were alkali-denatured with an equal volume of 2 M NaOH. It is known that the alkali-denatured RFP chromophore converts to a GFP-like one, with extinction coefficient 44,000 M^−1^ cm^−1^ at 452 nm under these conditions. Absorption, emission, and excitation spectra were recorded in PBS. Path lengths for each well were calculated ratiometrically using the difference in absorbance of PBS at 900 nm and 998 nm. Based on the absorption spectra of native proteins and the concentration determination of alkali-denatured proteins, molar extinction coefficients were calculated. For determination of quantum yields, the integrated fluorescence intensity of mutants of interest was compared with that of equally absorbing samples of mCherry and DsRed (quantum yields 0.22 and 0.80, respectively) with excitation at 550 nm.

### pH Studies and pKa Measurements

pH titrations were performed using a range of buffers from pH 2.0 to 9.5. Proteins were diluted into these buffers to a concentration of 5–10 µM. Fluorescence and absorption scans were taken at each pH value using a Tecan Safire2 plate reader. The Henderson-Hasselbach equation was used to calculate the pKa for each protein.

### Maturation Studies

To start, 30 mL cultures were inoculated with frozen cell stocks of *E. coli* BL21-Gold(DE3) containing the gene of interest in a pET11-a vector. After growing for 2–3 h at 37°C with shaking, cultures were induced with 5 mM IPTG, then capped and sealed to create an anaerobic environment. Proteins were expressed under these anaerobic conditions for 3–4 h at room temperature. After expression, cells were harvested by centrifugation and resuspended in 1.8 mL deoxygenated lysis buffer (50 mM Tris-HCl, pH 8.0, 300 mM NaCl, 5 mM imidazole, 1X CelLytic B, 1 mg/mL lysozyme, and 25 U/mL benzonase nuclease (Novagen)). Resuspended cell lysates were sealed from the air, then incubated at room temperature for 30–40 min without shaking to allow for complete cell lysis. After centrifugation, clarified lysates were recovered and proteins were quickly purified by Ni-NTA affinity chromatography at 4°C. Absorption and emission spectra were recorded with a Tecan Safire2 plate reader. Spectroscopic data collection for maturation studies was performed at 28°C for all proteins studied here. All experiments were performed in triplicate.

### Crystallography

A very large purple crystal of mPlum-E16P was grown in 0.3 µL×0.3 µL sitting drops with a precipitant solution of 200 mM MgCl, 100 mM sodium cacodylate, pH 6.5, and 50% (v/v) polyethylene glycol 200. This large rhomboidal crystal had approximate dimensions 0.4 mm×0.5 mm×0.1 mm. Bluish crystalline chunks of mPlumAYC-E16A were grown in sitting drops with 0.3 µL protein solution and 0.3 µL of the same precipitant used to crystallize mPlum-E16P; approximate dimensions were 0.05 mm×0.10 mm×0.08 mm. Bright yellow cubes of mPlumAYC were grown in sitting drops with 0.3 µL protein solution and 0.3 µL of a precipitant solution consisting of 200 nM sodium cacodylate, 100 mM Tris-HCl, pH 8.5, and 30% (w/v) polyethylene glycol 4000. These cubes had approximate dimensions of 0.05 mm×0.05 mm×0.05 mm. Crystals of mPlumAYC-E16P were grown but these did not display significant diffraction at high enough resolution to produce a dataset of sufficient quality to lead to a solvable structure.

All datasets were collected at the Stanford Synchrotron Radiation Lightsource beamline 12-2. IPMOSFLM [36] was used for integration and SCALA [37] was used for merging and scaling. All datasets collected were solved by molecular replacement using PHASERMR [38]. The search model used consisted of the PDB coordinates from mPlum (2QLG [21]) with the chromophore removed. Refinement was accomplished using REFMAC5 [39], [40] and PHENIX [41]. PHENIX was used specifically for refinement of atomic occupancies. Model building was done with COOT [42], wherein water molecules were added manually when they were within H-bonding distance of other heteroatoms (2.3–3.5 Å) and had peaks in the F_o_ – F_c_ map of greater than 3.5 σ. In addition, water molecules were removed when they had equivalent isotropic B-factors greater than 60–80 Å^2^. During generation of R-factors, 5% of data was excluded for cross-validation with an R_free_ value. Crystallographic R-factors were calculated in the standard fashion (R = ∑ |F_obs_−F_calc_|/∑ F_obs_). For all crystal structures, the final refinement steps were carried out with 20 translation-libration-screw (TLS) groups per protein molecule [43]. TLS groups were identified automatically by using the TLS Motion Determination web server [44]. Riding hydrogens were included in the refinement of all structures for non-water molecules consistent with the PHENIX online documentation for crystallographic refinement [http://www.phenix-online.org/documentation/]. The library file for the chromophore was built based on the CH6 chromophore deposited in the Hetero-compound Information Centre - Uppsala (HIC-Up) online database.

### Accession Numbers

The atomic coordinates and structure factors of mPlumAYC, mPlumAYC-E16A and mPlum-E16P have been deposited in the Protein Data Bank (PDB) under accession codes 4H3N, 4H3M, and 4H3L, respectively.

## Supporting Information

Figure S1 **pH affects the absorption spectra of various RFPs.** All spectra are normalized to the 280 nm absorbance peak. Heavy green and blue traces represent spectra taken at pH 7.0 and 9.5, respectively. All remaining black traces are separated from each other by 0.5 pH units (specifically: pH 7.5, 8.0, 8.5, and 9.0).
(TIF)

Figure S2 **pH profiles of mPlum and its mutants.** Top row: integrated green fluorescence intensity between 510 nm and 600 nm; bottom row: integrated red fluorescence intensity between 590 nm and 700 nm. Data were fit with a Henderson-Hasselbalch model, which was used to determine chromophore pKa’s.
(TIF)

Figure S3 **Red fluorescence level in mPlumAYC E16 mutants.** Integrated red fluorescence intensity between 590 nm and 700 nm with excitation at 570 nm was measured for one to six samples of each mPlumAYC E16 point mutant. Shown are the average integrated fluorescence intensities with error bars representing ±1 standard deviation. The control sample contained an empty protein expression plasmid.
(TIF)

Figure S4 **Maturation experiments with recovery mutants.** All spectra are normalized to the 280 nm absorbance peak. Heavy black and blue traces represent the beginning (t = 0 h) and end (t = 20 h) of the maturation experiment, respectively. The time between each gray or black trace is 1.0 h. Arrows indicate the primary direction of peak movement during maturation. Each heavy red trace indicates the point in time when the 410 nm absorbance peak reached its maximum level during the course of maturation. Black traces occur before the 410 nm peak reaches its maximum level; gray traces occur after the maximum. Selected regions of each panel (insets) are magnified to illustrate the revelation of a more pronounced peak at 410 nm during maturation at pH 9.5.
(TIF)

Table S1 **Crystallography data.** Values in parenthesis are statistics for the highest resolution shell of data. *Although this dataset displays a high value for R_merge_, options to rescale at higher resolution were limited. For example, rescaling at 2.0 Å results in an R_merge_ of 21.5% and rescaling at 5.5 Å results in an R_merge_ of 17.1%. Notably, R_p.i.m._ at the given resolution (1.75 Å) is a much lower value, at 15.5%.
(DOCX)
