# Supplementary figures and images for: Recovery of Red Fluorescent Protein Chromophore Maturation Deficiency through Rational Design
Source: PLoS One. 2012 Dec 20;7(12):e52463. doi: 10.1371/journal.pone.0052463 (PMC3527499; doi:10.1371/journal.pone.0052463)

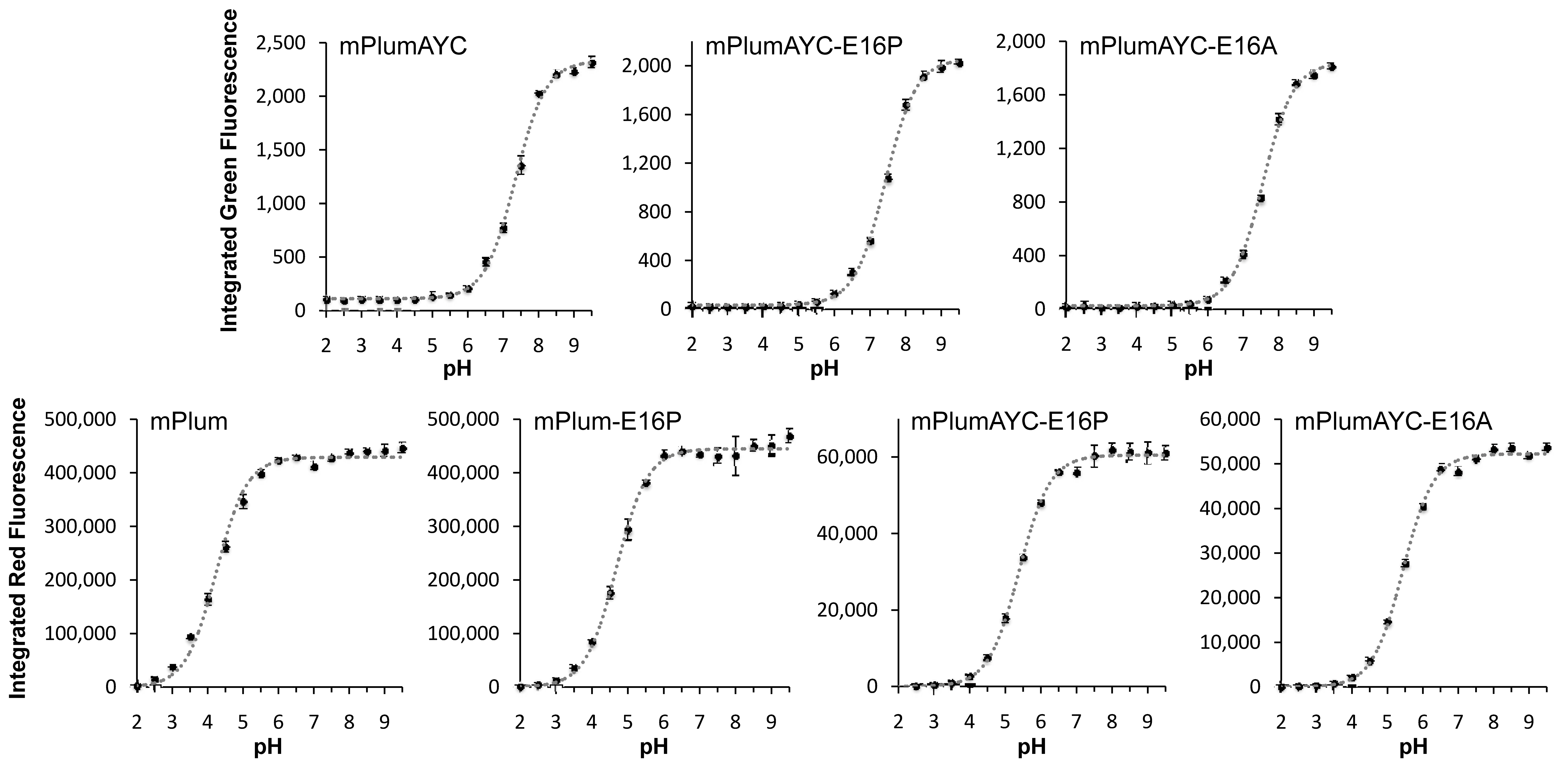

Supplement: Figure S2 — pH profiles of mPlum and its mutants. Top row: integrated green fluorescence intensity between 510 nm and 600 nm; bottom row: integrated red fluorescence intensity between 590 nm and 700 nm. Data were fit with a Henderson-Hasselbalch model, which was used to determine chromophore pKa’s. (TIF) [file pone.0052463.s002.tif]

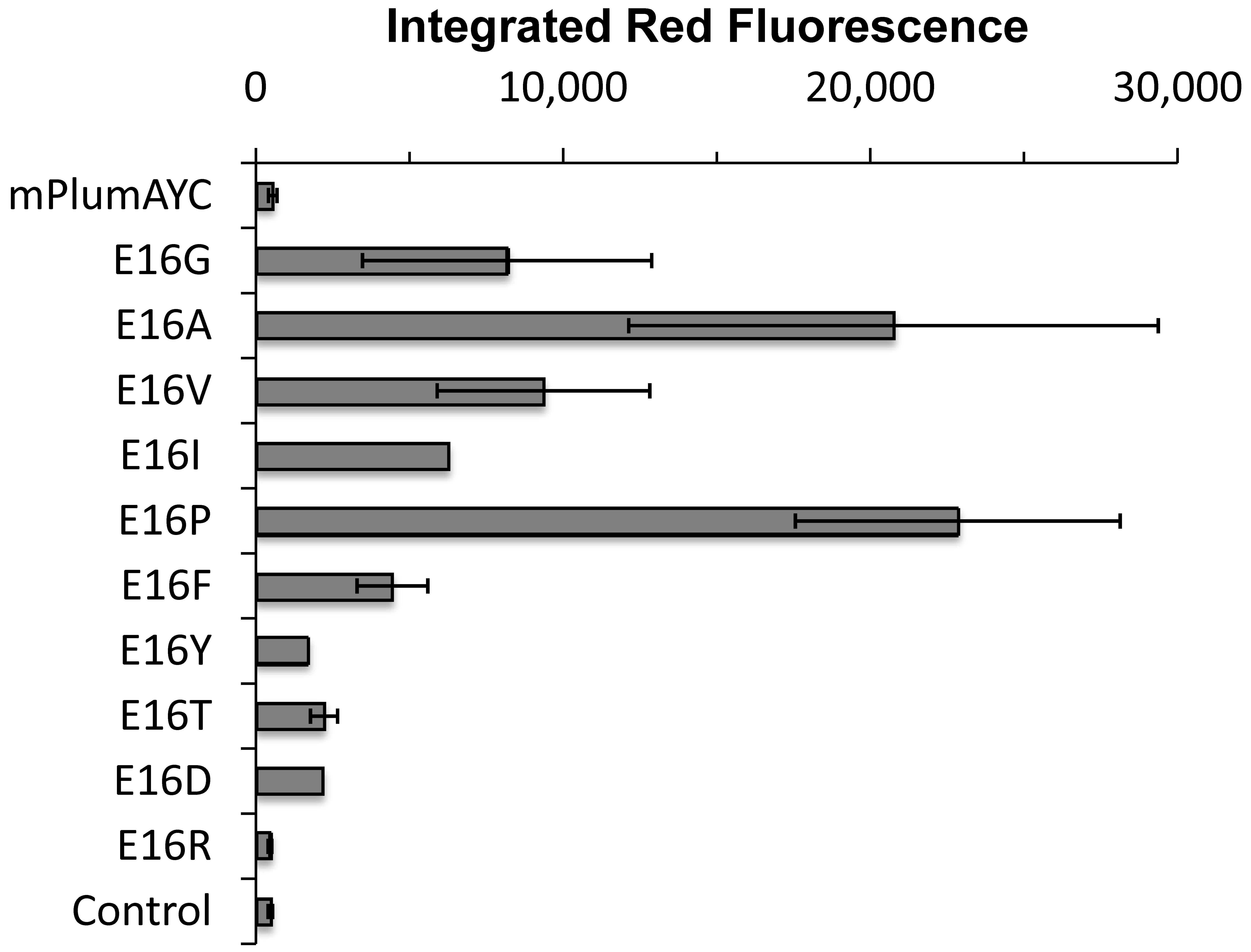

Supplement: Figure S3 — Red fluorescence level in mPlumAYC E16 mutants. Integrated red fluorescence intensity between 590 nm and 700 nm with excitation at 570 nm was measured for one to six samples of each mPlumAYC E16 point mutant. Shown are the average integrated fluorescence intensities with error bars representing ±1 standard deviation. The control sample contained an empty protein expression plasmid. (TIF) [file pone.0052463.s003.tif]

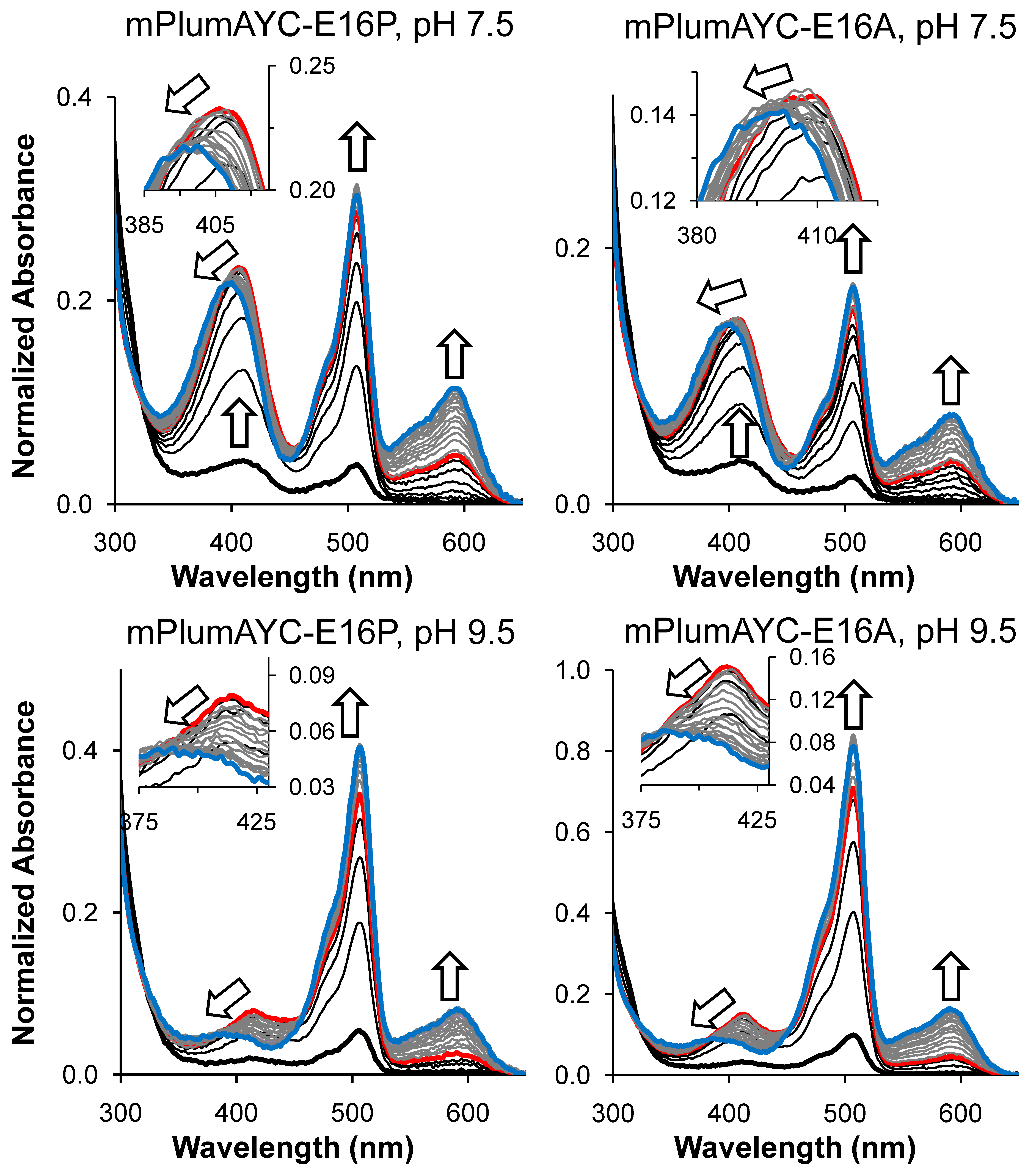

Supplement: Figure S4 — Maturation experiments with recovery mutants. All spectra are normalized to the 280 nm absorbance peak. Heavy black and blue traces represent the beginning (t = 0 h) and end (t = 20 h) of the maturation experiment, respectively. The time between each gray or black trace is 1.0 h. Arrows indicate the primary direction of peak movement during maturation. Each heavy red trace indicates the point in time when the 410 nm absorbance peak reached its maximum level during the course of maturation. Black traces occur before the 410 nm peak reaches its maximum level; gray traces occur after the maximum. Selected regions of each panel (insets) are magnified to illustrate the revelation of a more pronounced peak at 410 nm during maturation at pH 9.5. (TIF) [file pone.0052463.s004.tif]
