# Supplementary material for: Recovery of Red Fluorescent Protein Chromophore Maturation Deficiency through Rational Design
Source: PLoS One. 2012 Dec 20;7(12):e52463. doi: 10.1371/journal.pone.0052463 (PMC3527499; doi:10.1371/journal.pone.0052463)
Supplement: Table S1 — Crystallography data. Values in parenthesis are statistics for the highest resolution shell of data. *Although this dataset displays a high value for Rmerge, options to rescale at higher resolution were limited. For example, rescaling at 2.0 Å results in an Rmerge of 21.5% and rescaling at 5.5 Å results in an Rmerge of 17.1%. Notably, Rp.i.m. at the given resolution (1.75 Å) is a much lower value, at 15.5%. (DOCX) [file pone.0052463.s005.docx]

**Table S1.** Crystallography data.

|  | **mPlum-E16P** | **mPlumAYC** | **mPlumAYC-E16A** |
| --- | --- | --- | --- |
| Resolution (Å) | 33.9–1.65 | 37.9–1.75 | 40.3–2.0 |
| Space group | P2_1_2_1_2_1_ | P22_1_2_1_ | P2_1_2_1_2_1_ |
| Observations | 178,646 | 74,017 | 61,173 |
| Unique observations | 53,773 | 22,710 | 23,844 |
| R_merge_ (%) | 8.2 (51.1) | 22.9* (38.7) | 10.3 (15.0) |
| Completeness (%) | 97.9 (97.5) | 95.5 (99.9) | 92.2 (95.1) |
| *I/σI* | 7.2 (1.7) | 3.5 (2.1) | 6.0 (4.4) |
| *R_work_/R_free_* (%) | 19.0 / 22.7 | 22.1 / 28.1 | 21.5 / 27.4 |
| Molecules/asymmetric unit | 2 | 1 | 2 |
| Unit cell dimensions (Å) | *a* = 61.2 | *a* = 38.7 | *a* = 61.2 |
|  | *b* = 76.9 | *b* = 61.6 | *b* = 64.7 |
|  | *c* = 95.8 | *c* = 96.3 | *c* = 94.8 |
|  | *α* = *β* = *γ* = 90° | *α* = *β* = *γ* = 90° | *α* = *β* = *γ* = 90° |

Values in parenthesis are statistics for the highest resolution shell of data.

***** Although this dataset displays a high value for R_merge_, options to rescale at higher resolution were limited. For example, rescaling at 2.0 Å results in an R_merge_ of 21.5% and rescaling at 5.5 Å results in an R_merge_ of 17.1%. Notably, R_p.i.m._ at the given resolution (1.75 Å) is a much lower value, at 15.5%.
